# Safety and patient-reported outcomes of multimodal PSMA PET/MRI-guided prostate biopsy: results from the prospective multicenter DEPROMP trial

**DOI:** 10.1007/s00345-026-06767-6

**Published:** 2026-09-17

**Authors:** Clarissa Julia Schmidt, Tim Reichert, Florian C. Gaertner, Barbara Kreppel, Markus Essler, Julian A. Luetkens, Ulrike Attenberger, Michael Anspach, Carsten-Henning Ohlmann, Stefan Hauser, Jörg Ellinger, Manuel Ritter, Philipp Krausewitz

**Affiliations:** 1 Department of Urology and Paediatric Urology, University Hospital Bonn, Bonn, North Rhine-Westphalia Germany; 2 Department of Nuclear Medicine, University Hospital Bonn, Bonn, North Rhine-Westphalia Germany; 3 Department of Diagnostic and Interventional Radiology, University Hospital Bonn, Bonn, North Rhine-Westphalia Germany; 4 Department of Radiology and Nuclear Medicine, Medical University of Vienna, Vienna, Austria; 5 Department of Urology, Johanniter Hospital Bonn, Bonn, North Rhine-Westphalia Germany; 6 Department of Urology, GFO Hospital Troisdorf, Troisdorf, North Rhine-Westphalia Germany

**Keywords:** Prostate biopsy, PSMA-PET/CT, MRI, Patient reported outcome, Adverse effects

## Abstract

**Purpose:**

Multiparametric MRI and PSMA PET/CT have improved prostate cancer detection and targeting. While MRI-guided biopsy is well established, data on the procedural safety and patient-reported outcomes (PROs) of multimodal MRI- and PSMA-guided biopsy remain limited. We prospectively evaluated adverse events (AEs) and PROs following multimodal prostate biopsy.

**Methods:**

In the prospective, multicenter DEPROMP trial, 230 biopsy-naïve men with suspected prostate cancer underwent systematic, MRI-targeted, and PSMA PET/CT-targeted transrectal biopsy. AEs were assessed longitudinally and graded using the Clavien–Dindo classification. Associations between biopsy core number and AEs were analyzed. PROs were evaluated at 6 months using structured interviews. Multivariable analyses assessed predictors of PROs.

**Results:**

AEs were infrequent post-biopsy (94.8% without symptoms) and declined over time, with 87.4% symptom-free at 6 months. Most AEs were low-grade (Clavien–Dindo 1–2). Infectious complications were rare. At 6 months, 42.6% of patients reported unchanged and 22.1% improved quality of life, whereas 29.5% reported deterioration. Quality-of-life changes were more frequently attributed to cancer diagnosis (49.6%) than to biopsy-related AEs (1.7%). Cancer diagnosis was independently associated with worse quality of life (OR 12.94, *p* < 0.001), while AEs were not.

**Conclusion:**

Multimodal MRI- and PSMA-guided prostate biopsy was associated with low morbidity, high patient acceptance, and favorable PROs. Quality-of-life changes were more frequently attributed to cancer diagnosis than to biopsy-related AEs. These findings provide patient-centered evidence supporting contemporary image-guided prostate cancer diagnostic pathways and highlight the importance of addressing psychological burden during patient counseling.

## Introduction

Prostate cancer is one of the most commonly diagnosed malignancies in men worldwide, and histological confirmation remains essential for diagnosis. Transrectal systematic biopsy has long been the standard approach. However, limited detection accuracy and concerns regarding infectious and noninfectious complications have driven the development of more refined strategies [1]. Multiparametric MRI has substantially improved lesion localization and is now integrated into diagnostic pathways, with MRI-guided biopsy showing higher detection rates of clinically significant cancer without a clinically meaningful increase in morbidity compared with systematic biopsy alone [2–7]. In parallel, PSMA-PET/CT has emerged as a complementary imaging modality with high lesion conspicuity that may further enhance targeting accuracy [8, 9]. While MRI-guided and fusion biopsy techniques have demonstrated favorable safety profiles in prospective studies [10], data on the procedural safety of combined MRI- and PSMA-PET/CT–informed biopsy strategies remain limited. Most PSMA-PET/CT studies focus on diagnostic performance rather than adverse events (AEs) or patient experience following additional image-guided sampling [11–13]. Moreover, longitudinal assessments of biopsy-related morbidity at predefined timepoints are scarce, despite their importance for patient counseling and shared decision-making. Patient-reported outcomes (PROs) constitute an additional but underrepresented dimension. Existing studies often rely on single timepoint assessments, heterogeneous instruments, or retrospective designs, limiting comprehensive evaluation of the postprocedural course [14]. Previous psycho-oncological studies suggest that anxiety peaks during diagnostic uncertainty and declines after diagnostic clarification [15]. Understanding the relative impact of procedure-related effects and cancer diagnosis is therefore important when evaluating contemporary biopsy pathways.

While diagnostic performance of MRI-targeted and PSMA-informed biopsy pathways has been increasingly investigated, evidence regarding procedural morbidity and patient-reported outcomes remains scarce. The integration of multiple imaging modalities may increase sampling extent through additional biopsy targets and more complex diagnostic pathways [16, 17]. As advanced imaging becomes more deeply integrated into prostate cancer diagnostics, understanding patient experience and procedure-related burden is essential for patient counseling and clinical decision-making. Therefore, this secondary analysis of the prospective DEPROMP trial evaluated prospectively collected adverse events and patient-reported outcomes following multimodal MRI- and PSMA-guided prostate biopsy. By assessing morbidity at predefined timepoints and systematically capturing quality-of-life data, this study provides patient-centered evidence supporting contemporary image-guided prostate cancer diagnostic pathways.

## Methods

### Study design and setting

This study represents a secondary analysis of the prospective, open-label, multicentre DEPROMP trial, the protocol and primary analyses of which have been published previously [6, 18]. Whereas the parent DEPROMP trial primarily evaluated the diagnostic and therapeutic impact of combined MRI- and PSMA-informed biopsy, the present analysis focuses on prospectively collected adverse-event data and patient-reported outcomes following biopsy. For the present analysis, adverse-event severity was additionally categorized according to the Clavien–Dindo classification to provide a standardized assessment of procedural morbidity.

### Participants

All 230 men included in this analysis were enrolled in the DEPROMP trial [6, 18]. Eligible participants were biopsy-naïve men with suspected prostate cancer who were suitable for multiparametric MRI, PSMA-PET/CT, and transrectal fusion biopsy [6]. Men with contraindications to imaging or biopsy or inability to provide informed consent were excluded.

### Imaging procedures

All participants underwent multiparametric MRI and PSMA-PET/CT prior to biopsy according to standardized workflows [6]. Imaging findings were interpreted according to PI-RADS v2.1 and miTNM criteria and used for biopsy planning. In this analysis, imaging served for procedural planning and was not assessed for diagnostic performance.

### Biopsy procedure

After imaging, all men underwent triple-modality transrectal biopsy in a single session: (i) 12-core systematic biopsy, (ii) MRI–ultrasound fusion-targeted biopsy of PI-RADS 3–5 lesions, and (iii) PSMA-PET/CT–ultrasound fusion-guided biopsy of 1–3 miPSMA-positive regions. Procedures were performed under intravenously applied antibiotic prophylaxis with 2 g ceftriaxone, transrectal cleansing with iod and local analgesia using transrectal Instillagel as well as mepivacaine by trained urologists to minimize variability, reflecting the standard-of-care in 2019 prior to widespread adoption of the transperineal approach [6, 18]. Peri-procedural anticoagulation management followed institutional standards. Core numbers varied according to imaging findings and were recorded for exploratory analyses of associations with AEs.

### Adverse event documentation

Predefined biopsy-related AEs were prospectively recorded at study visits 3 (t1) and 4 (t2) according to the DEPROMP protocol [6]. Additional AE information was obtained during the six-month telephone follow-up (t3). For the present secondary analysis, the collected AE data were subsequently categorized according to the Clavien–Dindo classification. Events were categorized using predefined criteria (orthostatic symptoms, urinary retention, hematuria, hematospermia, infectious complications, rectal bleeding, symptomatic prostatitis, and other procedure-related symptoms For the present secondary analysis, the prospectively collected AE data were retrospectively categorized according to the Clavien–Dindo classification.Serious AEs were defined according to regulatory standards and the DEPROMP protocol as events requiring hospitalization, life-threatening events, or events causing significant functional impairment. In a predefined subanalysis, the association between anticoagulation therapy and the occurrence of AEs was explored.

### Patient-reported outcomes

PROs were assessed using structured telephone interviews at six months post-biopsy (t3); 218/230 patients (94.8%) completed this follow-up. Patients reported perceived changes in quality of life and their perceived attribution to biopsy-related AEs or cancer diagnosis.Patients were asked to report (i) perceived changes in overall quality of life compared with the previous year, (ii) the perceived cause of any change (biopsy-related AEs vs. cancer diagnosis), and (iii) willingness to undergo repeat biopsy under local anesthesia if clinically indicated. Categorical response options were predefined and analyzed without post hoc modification. Missing responses were reported as separate categories for descriptive purposes but excluded from inferential analyses. To address potential treatment-related confounding during follow-up, subgroup analyses compared surgically treated cancer, non-surgically treated cancer, and negative biopsy groups. In addition, multivariable logistic regression models were applied to evaluate independent associations between clinical variables (including cancer diagnosis and AEs) and PRO endpoints, including deterioration in quality of life and willingness to undergo repeat biopsy.

### Statistical analysis

Descriptive statistics summarized AE frequencies, PRO distributions, and Clavien–Dindo classifications as counts and percentages. Missing data were reported descriptively and excluded from inferential analyses; no imputation was performed. Associations between biopsy core number and AEs were examined using contingency tables, with core counts grouped as 4–15, 17–21, and 24–27 and AEs categorized as present or absent. Fisher’s exact test was used to assess associations at each timepoint. PRO group comparisons used Fisher’s exact test with Monte Carlo simulation where appropriate because of small expected cell counts. Subgroup analyses evaluated PROs according to cancer diagnosis and surgical treatment status. Multivariable logistic regression evaluated associations of surgery, cancer diagnosis, and AEs with worse quality of life and willingness to undergo repeat biopsy, with results reported as odds ratios (ORs) and 95% confidence intervals (CIs). Analyses were performed using R version 4.4.3.

## Results

Event rates varied across follow-up visits, peaking at the intermediate assessment and declining thereafter.

### Early adverse events (t1)

Immediately post-biopsy, AEs were rare: 94.8% reported no symptoms. Urinary retention requiring catheterization occurred in 2.6% and orthostatic symptoms in 1.3%; 1.3% of data were missing. No infectious complications were observed.

According to Clavien–Dindo classification, 94.8% had no classifiable event, 1.3% were grade 1, and 2.6% grade 2 (1.3% missing).

### Intermediate adverse events (t2)

At follow-up, AEs were more frequent. Hematuria lasting > 2 days was most common (23.5%). Hematospermia occurred in 6.1% and combined hematuria/hematospermia in 4.8%. Urinary retention was reported in 0.9%, symptomatic cystitis in 0.4%, and prolonged rectal bleeding in 0.4%. One patient (0.4%) required hospitalization for hematuria with urinary retention and continuous bladder irrigation. Overall, 61.3% reported no AEs; 2.2% of data were missing.

According to Clavien–Dindo classification, 61.3% had no event, 34.8% grade 1, and 1.7% grade 2 (2.2% missing).

### Late adverse events (t3)

At six months, AEs had markedly declined: 87.4% reported no symptoms. Hematospermia and hematuria > 2 days each occurred in 2.6%, combined hematuria/hematospermia in 1.7%, and symptomatic prostatitis in 0.4%. Twelve patients (5.2%) did not complete the six-month telephone follow-up, resulting in 218 patients with available t3 data. According to Clavien–Dindo classification, 87.4% had no event, 7.0% grade 1, and 0.4% grade 2 (5.2% missing).

### Biopsy core number and adverse events

**Fig. 1:**
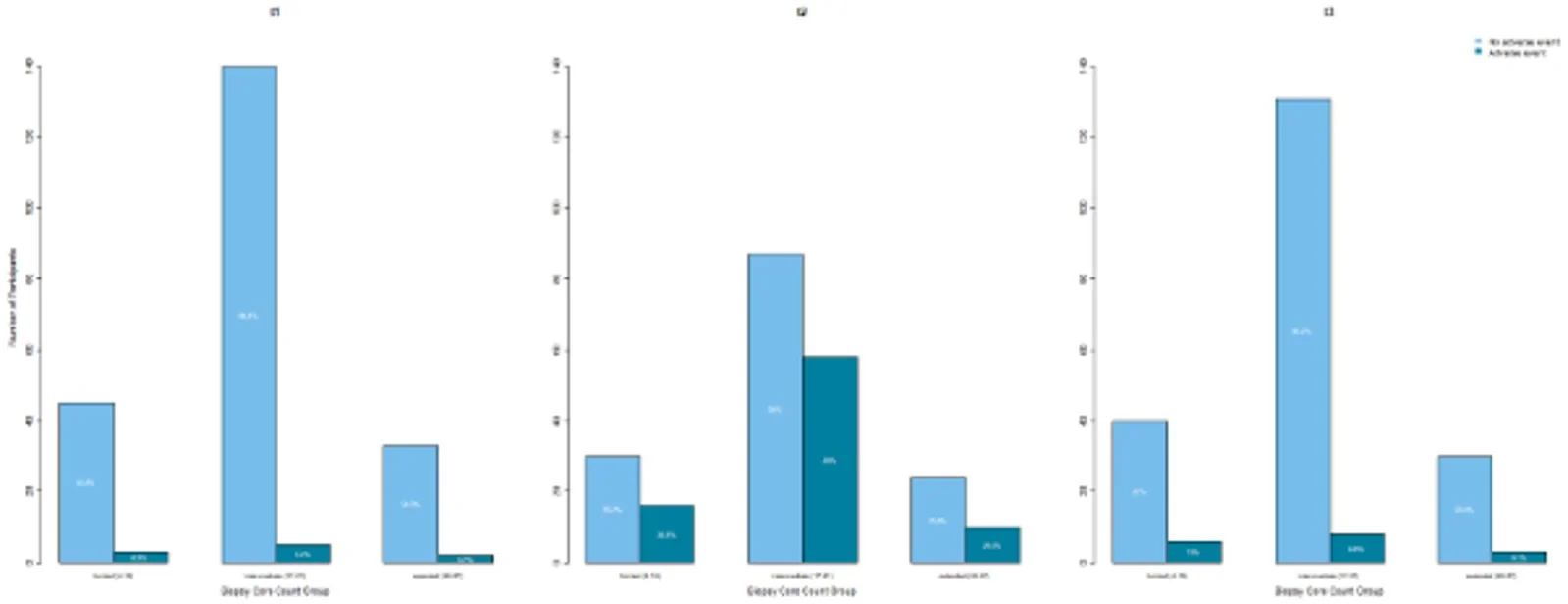
Adverse events by biopsy core count at t1, t2, and t3. Combined bar plots illustrating the number of participants with and without AEs across biopsy sampling groups (4–15, 17–21, and 24–27 cores) at the three predefined follow-up timepoints t1, t2, and t3. Light blue bars correspond to participants without AEs, and dark turquoise bars to those with AEs. Missing values were excluded (t1: *n* = 5; t2: *n* = 5; t3: *n* = 12). The y-axis was harmonized across panels to enable direct visual comparison

Across all biopsy core count categories, adverse event rates were comparable at each follow-up timepoint. No association between biopsy core number and adverse events was observed at t1 (*p* = 0.57), t2 (*p* = 0.50), or t3 (*p* = 0.24). Likewise, no overall association was observed across all timepoints (*p* = 0.76) (Fig. 1).

### Patient-reported outcomes at 6 months

At 6 months, 42.6% of patients reported no change in health-related quality of life and 0.4% reported being “about the same as before.” Improvement was reported by 22.1% (1.7% much better, 20.4% somewhat better), whereas 29.5% reported deterioration (27.8% somewhat worse, 1.7% much worse). Missing responses accounted for 5.2%.

Regarding perceived causes of change, 49.6% of patients attributed them to the cancer diagnosis, 1.7% to biopsy-related AEs, and 0.4% to a combination of both. Overall, 43.0% reported no change, and 5.2% did not respond.

Willingness to undergo repeat biopsy under local anaesthesia remained high. A total of 85.7% indicated they would agree to a repeat procedure if clinically indicated, while 9.1% would decline. Missing responses accounted for 5.2%.

**Fig. 2:**
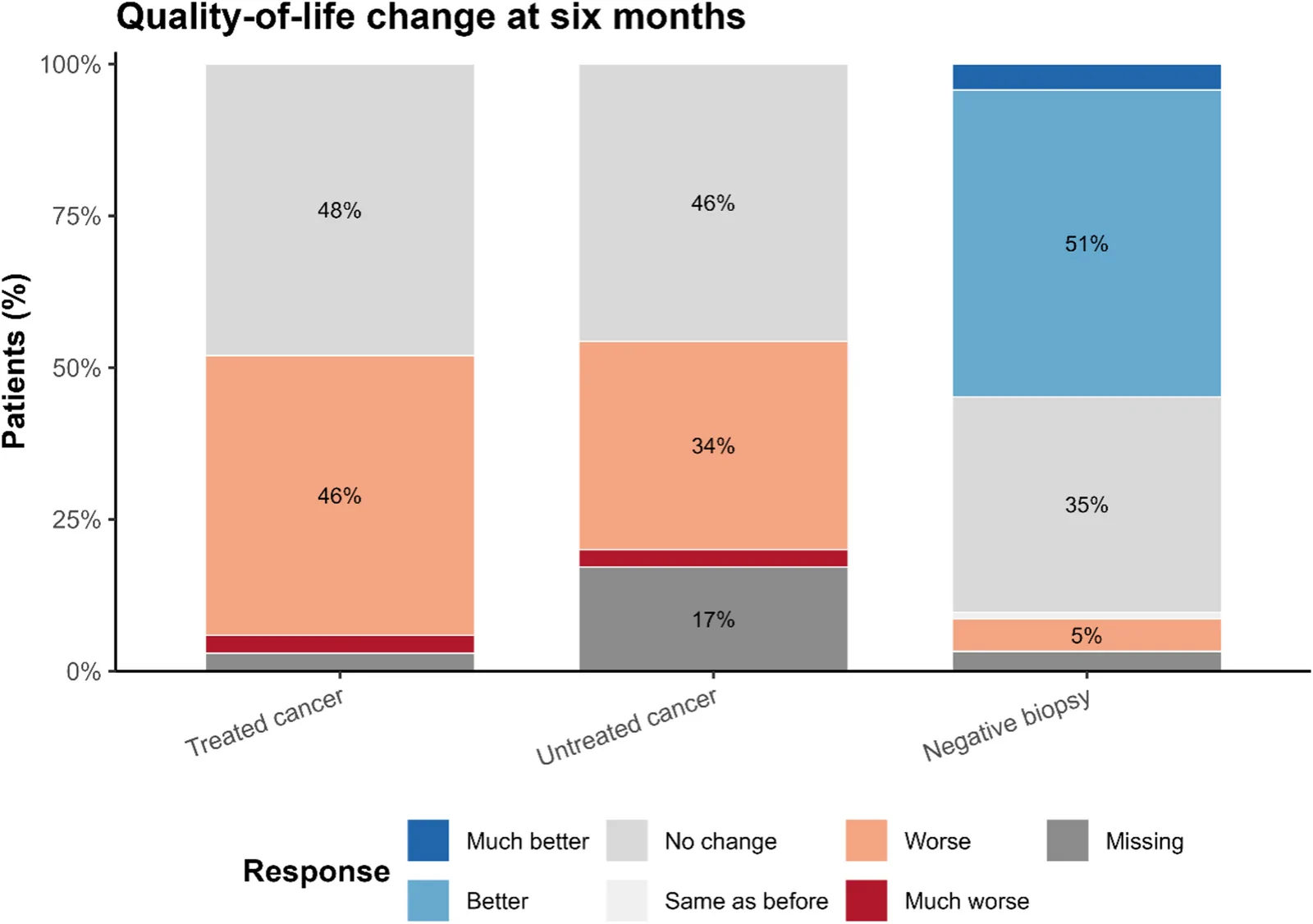
Distribution of patient-reported quality-of-life change at six months by clinical subgroup. Bars represent proportions within each subgroup. Response categories are shown as originally recorded, including missing responses

Overall, 137/230 patients (59.6%) were diagnosed with prostate cancer and 106/230 (46.1%) underwent surgery during follow-up. When comparing patients with versus without surgery, quality-of-life outcomes differed significantly (*p* < 0.001), whereas perceived causes of change (*p* = 0.115) and willingness to undergo repeat biopsy (*p* = 0.109) did not. Subgroup analyses across clinical categories, including surgically treated cancer, untreated cancer, and negative biopsy, showed differences in quality-of-life change (*p* < 0.001) (Fig. 2) and perceived causes (*p* = 0.036), but not in willingness for repeat biopsy (*p* = 0.212). However, among patients with a cancer diagnosis, no significant differences were observed between surgically and non-surgically treated patients for quality-of-life change (*p* = 0.856), perceived cause of change (*p* = 0.071), or willingness to undergo repeat biopsy (*p* = 0.230).In multivariable analysis, a cancer diagnosis was strongly associated with worse quality of life (odds ratio [OR] 12.94, 95% CI 4.45–44.14; *p* < 0.001), whereas surgery (OR 1.46, 95% CI 0.64–3.34; *p* = 0.366) and AEs (OR 1.35, 95% CI 0.70–2.64; *p* = 0.369) were not. Willingness to undergo repeat biopsy was not associated with surgery or cancer diagnosis but was significantly lower in patients with AEs (OR 0.22, 95% CI 0.07–0.59; *p* = 0.005).

### Anticoagulation and overall adverse events

AEs occurred across all anticoagulation categories and were predominantly low-grade (Clavien–Dindo 1–2). Although numerically more frequent among patients receiving anticoagulation, no increase in clinically significant complications was observed.

## Discussion

In this prospective multicenter analysis from the DEPROMP cohort, multimodal prostate biopsy incorporating MRI and PSMA PET/CT was associated with low morbidity and favorable patient-reported outcomes. These findings extend previous high-quality data showing that MRI-guided biopsy does not substantially increase morbidity compared with systematic biopsy alone [4] and support the safe implementation of contemporary multimodal imaging-based biopsy strategies [10–12]. Importantly, prospective data on AEs and patient-reported outcomes following combined MRI and PSMA PET/CT-guided biopsy remain limited. Our findings therefore provide patient-centered evidence supporting contemporary image-guided prostate cancer diagnostic pathways. An additional finding was the absence of an association between biopsy sampling extent and AEs across all timepoints. Although multimodal imaging may increase the number of biopsy targets, higher core numbers were not associated with increased morbidity. These findings raise the hypothesis that procedural quality may be more relevant than sampling extent for complication risk [19]. However, because all participants underwent the same combined systematic, MRI-targeted, and PSMA-targeted biopsy protocol and adverse events were infrequent, the independent contribution of PSMA-targeted biopsy cores cannot be determined, and this exploratory observation requires confirmation in comparative studies. Nevertheless, our results and interpretation are consistent with emerging data supporting the safety of contemporary image-guided biopsy approaches [20]. A key strength of this study is the longitudinal assessment of AEs across three predefined timepoints. AEs declined substantially over time, with the majority of patients reporting complete resolution at six months and a high willingness to undergo repeat biopsy. This temporal pattern aligns with prospective data demonstrating rapid resolution of biopsy-related morbidity within weeks [21], although a small proportion of patients continued to report minor symptoms such as hematospermia at later follow-up [22].

Despite the favorable safety profile, limitations inherent to the transrectal approach must be acknowledged. Infectious complications remain clinically relevant due to rectal mucosal transgression [22, 23]. In our cohort, infectious events were rare, with no infections at t1 and only 0.4% cystitis at t2 and 0.4% prostatitis at t3. These rates are lower than those reported in previous prospective and population-based studies [22–24], potentially reflecting the prophylactic measures used in our cohort. Beyond infection, bleeding-related events remain common but are typically self-limiting [22], although population-based data confirm that urological complications may require hospital admission in a subset of patients [25].

AEs occurred across all anticoagulation categories without an increase in clinically significant complications. This is consistent with previous evidence showing that most biopsy-related complications are minor [22], that low-dose aspirin does not increase bleeding severity [26], and that anticoagulation is mainly associated with minor bleeding events [27]. Together with the overall low complication rates observed in this cohort, these findings support the favorable safety profile of contemporary multimodal image-guided prostate biopsy pathways.

Another important observation was that, at the six-month interview, patients more frequently attributed perceived quality-of-life changes to the cancer diagnosis than to biopsy-related adverse events. However, these findings should be interpreted cautiously. Quality of life was assessed only once using a structured study-specific interview without baseline assessment or a validated extended patient-reported outcome measure. Consequently, patients’ attribution reflects their perception at the time of interview and may have been influenced by recall bias, the interview framework, and downstream clinical management, including treatment received after cancer diagnosis. Therefore, these findings should be regarded as descriptive and hypothesis-generating rather than evidence of a causal relationship between biopsy, cancer diagnosis, and subsequent quality of life. Most patients nevertheless reported stable or improved quality of life, and biopsy-related adverse events were rarely perceived as the main contributor to impairment. This is consistent with previous reports indicating that biopsy-related symptoms are generally mild and well tolerated [21, 22], whereas clinically relevant complications remain uncommon but possible [25]. Patients more frequently attributed quality-of-life changes to the cancer diagnosis than to biopsy-related adverse events, an observation that is in line with previous studies showing that health-related quality of life may already be influenced by cancer diagnosis and treatment decision-making [28]. Taken together, our findings raise the hypothesis that the psychological burden associated with cancer diagnosis may contribute more to patients’ perceived quality of life than biopsy-related morbidity. However, this interpretation remains descriptive and requires confirmation using validated longitudinal patient-reported outcome measures. Previous studies suggest that anxiety peaks before biopsy and declines after diagnostic clarification [15, 29]. Together with our findings, this indicates that psychological burden may contribute more to patient experience than biopsy-related morbidity and highlights the importance of patient counseling [30]. The increasing use of advanced imaging in prostate cancer diagnostics has raised concerns regarding the procedural burden associated with additional targeted biopsy sampling. Our findings suggest that integration of MRI- and PSMA-based targeting strategies does not result in a clinically meaningful increase in patient-reported morbidity and is associated with high patient acceptance. These data may support patient counseling and shared decision-making when discussing contemporary image-guided biopsy pathways.

Several limitations should be acknowledged. First, all biopsies were performed using a transrectal approach, reflecting the clinical standard at protocol initiation in 2019. Since then, the transperineal approach has become the preferred biopsy technique and is recommended by current clinical guidelines [31]. Therefore, direct transferability of our findings to contemporary transperineal multimodal biopsy pathways should be made with caution. Nevertheless, the prospective assessment of adverse events and patient-reported outcomes provides valuable information on the safety and patient experience of combined MRI- and PSMA-informed biopsy strategies, for which prospective evidence remains limited. Second, the relatively low number of clinically significant AEs limits statistical power for subgroup analyses. Furthermore, because all patients underwent the same combined systematic, MRI-targeted, and PSMA-targeted biopsy protocol, the incremental morbidity attributable to PSMA-targeted biopsy cores could not be assessed. The exploratory analysis relating biopsy core number to adverse events should therefore be interpreted cautiously given the low adverse-event rate and limited statistical power. Third, PROs were assessed using a single structured study-specific interview 6 months after biopsy rather than validated patient-reported outcome instruments, and no baseline assessment was available [14]. Therefore, patients’ attribution of quality-of-life changes should be interpreted cautiously, as it may have been influenced by recall bias and downstream treatment during follow-up. Besides, safety assessment was restricted to predefined biopsy-related AEs and may therefore not capture unanticipated complications. Furthermore, the absence of a procedural comparator precludes direct comparison with alternative biopsy strategies and prevents assessment of the independent contribution of the individual biopsy components to adverse events and patient-reported outcomes. Finally, this predefined secondary analysis was not designed to establish causal relationships.

Strengths of this study include its prospective design, standardized data collection, and longitudinal assessment of AEs and PROs in a contemporary cohort undergoing multimodal imaging-guided biopsy. These data address a relevant gap, as evidence on procedure-related morbidity of combined MRI and PSMA PET/CT-guided biopsy remains limited. Importantly, prospective multicentre data integrating longitudinal adverse events and patient-reported outcomes in multimodal MRI- and PSMA-informed biopsy pathways remain scarce. Although the present findings were obtained using a transrectal approach, they provide a prospective benchmark for the safety and patient experience of multimodal image-guided biopsy strategies and may inform future studies evaluating contemporary transperineal approaches.

## Conclusion

In this prospective multicenter study, multimodal MRI- and PSMA-guided prostate biopsy was associated with low morbidity, high patient acceptance, and favorable patient-reported outcomes. Quality-of-life changes were more frequently attributed to cancer diagnosis than to biopsy-related AEs. These findings provide important patient-centered evidence supporting implementation of contemporary image-guided prostate cancer diagnostic pathways and highlight the importance of addressing the psychological impact of cancer diagnosis during patient counseling.

## Data Availability

All data generated or analyzed during this study are included in this published article. Further inquiries can be directed to the corresponding author.

## Abbreviations

AEs: Adverse events
MRI: Magnetic resonance imaging
OR: Odds ratio
PET/CT: Positron emission tomography combined with computed tomography
PROs: Patient reported outcomes
PSMA: Prostate specific membrane antigen

## Declarations

### Generative AI and AI-assisted technologies in the writing process

During the preparation of this work, the author, Clarissa Julia Schmidt, used chat.openai.com to check grammar and spelling, as she is a non-native speaker. After using this tool/service, the author reviewed and edited the content as needed and takes full responsibility for the content of the publication.
